# Virtual reality in restorative dentistry: a bibliometric analysis of research trends

**DOI:** 10.1038/s41405-025-00364-2

**Published:** 2025-09-02

**Authors:** Manal Matoug-Elwerfelli, Alaa Daud, Kamran Ali, Ahmed Abdou

**Affiliations:** 1 Department of Pre-Clinical Oral Sciences, College of Dental Medicine, QU Health, Qatar University, Doha, Qatar; 2 Department of Restorative Dentistry, Faculty of Dentistry, Universiti Malaya, Kuala Lumpur, Malaysia

**Keywords:** Oral diseases, Diseases

## Abstract

**Background:**

Virtual reality (VR) technologies are increasingly being adopted in dental education, particularly in restorative dentistry, due to their capacity to simulate realistic clinical scenarios and enhance student learning. However, despite the growing interest, the overall research landscape in this field remains unclear. This study aimed to conduct a bibliometric analysis to evaluate the most influential research contributions, publication trends, and collaborative patterns related to VR in restorative dentistry.

**Methods:**

An advanced search of the Web of Science Core Collection (WoS-CC) database was performed on 29th October 2024 using a combination of keywords and Medical Subject Headings (MeSH) terms relevant to VR and restorative dentistry. Only original research and review articles in English were included. Bibliometric parameters such as citation metrics, authorship, institutional affiliations, countries of origin, and keywords were extracted and analyzed using Bibliometrix (R) and VOSviewer software.

**Results:**

Out of 434 retrieved records, 62 articles met the inclusion criteria. A marked increase in publications was observed after 2019, with the USA, UK, and Netherlands emerging as the leading contributors. The most cited article received 73 citations, focusing on haptic simulators for motor skill acquisition. The *Journal of Dental Education*, the *European Journal of Dental Education*, and *BMC Medical Education* were among the most prolific journals. Despite global contributions, the analysis revealed limited interdisciplinary and international collaboration.

**Conclusion:**

This bibliometric study highlights the increasing research activity surrounding VR in restorative dentistry and its potential to transform dental education. While considerable progress has been made, further high-quality research and broader collaborative efforts are necessary to address existing gaps and fully harness the capabilities of VR and related immersive technologies in dental education.

## Introduction

Technological advancements, particularly in virtual and immersive reality have contributed to a growing acceptance and integration of modern technology into the undergraduate dental curriculum over the last decade [1, 2]. These immersive technologies augment the physical environment by integrating virtual elements, thereby offering innovative opportunities for both clinical practice and educational applications [3, 4]. Given that students entering dental programs are often highly motivated to develop hands-on skills, such technologies facilitate skill acquisition through structured and dynamic learning approaches [5, 6]. Examples of their successful integration within undergraduate teaching in multiple specialties include; oral & maxillofacial surgery [7], paediatric dentistry [8] and operative/restorative dentistry, endodontics, and prosthodontics [5, 9, 10]. This integration reflects the evolution of dental education, where traditional pre-clinical teaching methods are increasingly complemented by state-of-the-art digital tools to enrich the student learning experiences [3, 4].

A recent scoping review by Al Hamad et al. identified significant inconsistencies and fragmentation in how immersive reality technologies are described within the existing literature, highlighting the absence of a unified taxonomy framework [3]. Commonly reported terms included augmented reality (AR), augmented virtuality (AV), extended reality, mixed reality (MR), virtual reality (VR), and X reality. This observation was echoed by Abbas et al. who also noted the variability and complexity in terminology and a proposed a contemporary definition of VR, tailored to healthcare education as “A *3D computer-generated simulated environment that aims to replicate real-world or imaginary settings and interactions”* [11]. In the field of restorative dentistry, also known as cariology or operative dentistry, VR has gained considerable attention due to its ability to enhance student learning through realistic simulations [5]. For example, VR-based haptic simulators allow students to practice cavity preparations with precise tactile feedback, replicating the sensation of working on natural or typodont teeth. Additionally, virtual patient cases enable students to diagnose and plan restorative treatments based on interactive 3D models, improving their clinical reasoning and decision-making skills [12]. These simulations provide a risk-free platform for students to develop their hand-eye coordination, improve procedural accuracy, and refine their clinical decision-making skills before transitioning to patient care [13]. Additionally, VR-based training has been shown to increase student engagement and confidence while reducing anxiety associated with clinical procedures [14].

Bibliometric analyses aim to provide a comprehensive understanding of the status of published literature in a given field or topic through a qualitative and quantitative analysis [15, 16]. Within dentistry, bibliometric citation analyses have been of significant interest and undertaken in several topics and fields, such as; COVID-19 related to dentistry [17], early childhood caries [18], endodontics [19], tooth auto transplantation [20] and regenerative endodontics [21]. Given the increasing application and pedagogical value of VR in dental education, particularly in restorative dentistry, it is essential to systematically evaluate the existing body of research to understand its development, scope, and future directions. Conducting bibliometric analysis allows for a structured exploration of publication trends, influential authors, and collaborative networks within the field of VR in dentistry.

To the best of the authors’ knowledge, no bibliometric research has been published in relation to VR in restorative dentistry. Therefore, this study aimed to analyze the top most cited articles related to the utilization of VR within restorative dentistry, to identify the most cited authors, institutions, countries of origin, gain insightful characteristics of influential publications, citation trends, collaborative research patterns and to outline the scientific advancements in this field.

## Material and methods

### Database and search strategy

An advanced electronic search of Clarivate Analytics’ Web of Science ‘Core Collection’ (WoS-CC; http://www.webofknowledge.com) database was performed on 29th October 2024, with no restrictions related to the year of publication. The search strategy involved a combination of Medical Subject Headings (MeSH) and keywords, as follows; TS =(“virtual reality” OR “augmented reality” OR “virtual simulation” OR “haptic” OR “haptics” OR “haptic interfaces” OR “haptic technology” OR “haptic simulation” OR “haptic feedback” OR “haptic virtual reality” OR “haptic 3D virtual reality” OR “haptic and force feedback technology” OR “stereognosis” OR “mixed reality” OR “mixed-reality” OR “mixed realities” OR “extended reality” OR “metaverse”) AND TS= (“dentistry” OR “restorative dentistry” OR operative dentistry OR cariology OR “dental education” OR “dentistry students” OR “dental trainees”).

### Inclusion and exclusion criteria

The authors acknowledge the broad term of restorative/operative dentistry within the current literature, and thus studies related to VR, AR, MR and conventional direct operative procedures such as caries removal, onlays and inlays, were included. The aforementioned procedures require precise internal cavity geometry with specific angulation constraints, primarily depending on tactile feedback. On the contrary, studies focusing on extra-coronal crown preparations, dental anatomy and morphology, virtual articulators, and 3D digital study models were excluded. In terms of study design, original research and review articles were included. Editorial, conference abstracts, proceedings and communication letters were also excluded. The search was restricted to articles published in English language.

### Bibliometric analysis methods

The retrieved data were saved in Microsoft Excel as ‘Plain Text’ with Full Record and Cited References. Title and abstract screening were performed by two calibrated and independent authors (A.A. & M.M.E.). Discrepancies were resolved by consensus or by discussion with the third author (K.A.).

The following bibliometric parameters were extracted from the included articles WoS-CC database: total citations (TC), TC per year, normalized TC, year of publication, first and contributing authors, countries (based on the affiliation of the corresponding author), institutions (based on all authors) and journal of publication. The data were imported into the R environment (R package Bibliometrix version 3.1) for relevant statistical computing. VOSviewer (version 1.6.20) was used to create network analysis of the most frequent words.

## Results

### Search results

**Fig. 1:**
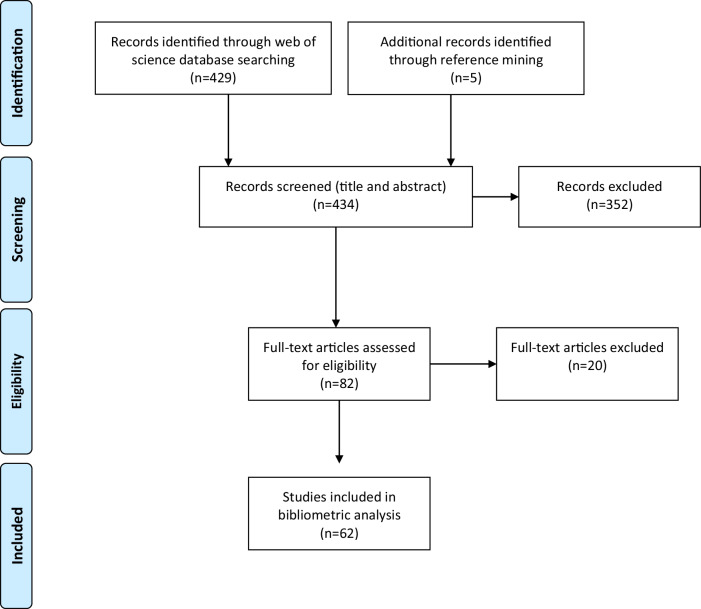
PRISMA flowchart outlining the articles selection process.

A comprehensive search on WoS-CC database retrieved a total of 429 articles, with an additional of 5 articles identified through reference mining. Following screening a total of 372 were excluded as they did not meet the inclusion criteria. Finally, a total of 62 articles were included in this bibliometric analysis as seen in Fig. 1 and Table 1.

**Table 1:** The ranking of the 62 included articles based on their Web of Science total citation count

Author (Year)	Title	Journal	Total citations^a^	Total citations per year	Normalized total citations
Al-Saud et al. (2017)	Feedback and motor skill acquisition using a haptic dental simulator	European Journal of Dental Education	73	9.13	3.12
Murbay et al. (2020)	Evaluation of the introduction of a dental virtual simulator on the performance of undergraduate dental students in the pre-clinical operative dentistry course	European Journal of Dental Education	63	12.60	1.49
De Boer et al. (2016)	Student performance and appreciation using 3D vs. 2D vision in a virtual learning environment	European Journal of Dental Education	62	6.89	1.00
Kwon et al. (2018)	Augmented reality in dentistry: a current perspective	Acta Odontologica Scandinavica	58	8.29	1.56
Ben Gal et al. (2011)	Preliminary assessment of faculty and student perception of a haptic virtual reality simulator for training dental manual dexterity	Journal of Dental Education	57	4.07	1.40
Nassar et al. (2020)	Computer simulation and virtual reality in undergraduate operative and restorative dental education: a critical review	Journal of Dental Education	56	11.20	1.33
Farronato et al. (2019)	Current state of the art in the use of augmented reality in dentistry: a systematic review of the literature	BMC Oral Health	56	9.33	2.00
Mirghani et al. (2018)	Capturing differences in dental training using a virtual reality simulator	European Journal of Dental Education	53	7.57	1.42
Eve et al. (2014)	Performance of dental students versus prosthodontics residents on a 3d immersive haptic simulator	Journal of Dental Education	51	4.64	1.00
Serrano et al. (2020)	First experiences with patient-centered training in virtual reality	Journal Of Dental Education	44	8.80	1.04
Vincent et al. (2020)	Contribution of haptic simulation to analogic training environment in restorative dentistry	Journal Of Dental Education	41	8.20	0.97
Thomas et al. (2001)	The design and testing of a force feedback dental simulator	Computer Methods and Programs In Biomedicine	40	1.67	1.00
Llena et al. (2018)	Implementation of augmented reality in operative dentistry learning	European Journal of Dental Education	39	5.57	1.05
Gottlieb et al. (2011)	Faculty impressions of dental students’ performance with and without virtual reality simulation	Journal of Dental Education	39	2.79	0.96
Urbankova et al. (2013)	A complex haptic exercise to predict preclinical operative dentistry performance: a retrospective study	Journal of Dental Education	30	2.50	1.50
Wierinck et al. (2007)	Expert performance on a virtual reality simulation system	Journal of Dental Education	29	1.61	1.00
De Boer et al. (2017)	The effect of force feedback in a virtual learning environment on the performance and satisfaction of dental students	Simulation In Healthcare-Journal of The Society for Simulation In Healthcare	28	3.50	1.20
Urbankova et al. (2011)	The use of haptics to predict preclinic operative dentistry performance and perceptual ability	Journal of Dental Education	26	1.86	0.64
De Boer et al. (2019)	The effect of variations in force feedback in a virtual reality environment on the performance and satisfaction of dental students	Simulation In Healthcare-Journal Of The Society For Simulation In Healthcare	24	4.00	0.86
Koo et al. (2015)	An initial assessment of haptics in preclinical operative dentistry training	Journal of Investigative and Clinical Dentistry	22	2.20	1.10
Dixon et al. (2021)	Re-defining the virtual reality dental simulator: demonstrating concurrent validity of clinically relevant assessment and feedback	European Journal of Dental Education	21	5.25	1.29
Al-Saud LM (2021)	The utility of haptic simulation in early restorative dental training: a scoping review	Journal of Dental Education	18	4.50	1.10
Dwisaptarini et al. (2018)	Effectiveness of the multilayered caries model and visuo-tactile virtual reality simulator for minimally invasive caries removal: a randomized controlled trial	Operative Dentistry	18	2.57	0.48
Ria et al. (2018)	A scoring system for assessing learning progression of dental students’ clinical skills using haptic virtual workstations	Journal of Dental Education	18	2.57	0.48
Espejo-Trung et al. (2015)	Development and application of a new learning object for teaching operative dentistry using augmented reality	Journal of Dental Education	18	1.80	0.90
Dzyuba et al. (2022)	Virtual and augmented reality in dental education: the good, the bad and the better	European Journal of Dental Education	16	5.33	1.78
Towers et al. (2022)	Combining virtual reality and 3d-printed models to simulate patient-specific dental operative procedures-a study exploring student perceptions	European Journal of Dental Education	15	5.00	1.67
San Diego et al. (2022)	Learning clinical skills using haptic vs. Phantom head dental chair simulators in removal of artificial caries: cluster-randomized trials with two cohorts’ cavity preparation	Dentistry Journal	14	4.67	1.56
Farag et al. (2022)	Impact of the haptic virtual reality simulator on dental students’ psychomotor skills in preclinical operative dentistry	Clinics and Practice	13	4.33	1.44
Koolivand et al. (2024)	Comparison of the effectiveness of virtual reality-based education and conventional teaching methods in dental education: a systematic review	BMC Medical Education	12	12.00	9.00
Rodrigues et al. (2022)	Usability, acceptance, and educational usefulness study of a new haptic operative dentistry virtual reality simulator	Computer Methods and Programs In Biomedicine	10	3.33	1.11
Osnes et al. (2021)	Investigating the construct validity of a haptic virtual caries simulation for dental education	BMJ Simulation & Technology Enhanced Learning	10	2.50	0.61
Kuchenbecker et al. (2017)	Evaluation of a vibrotactile simulator for dental caries detection	Simulation In Healthcare-Journal of The Society for Simulation In Healthcare	10	1.25	0.43
Kozhevnikov et al. (2013)	Egocentric versus allocentric spatial ability in dentistry and haptic virtual reality training	Applied Cognitive Psychology	10	0.83	0.50
Rodrigues et al. (2023)	Preclinical dental students self-assessment of an improved operative dentistry virtual reality simulator with haptic feedback	Scientific Reports	8	4.00	2.29
Tricio et al. (2022)	Students’ and tutors’ perceptions of a deliberate simulated practice using patient-specific virtual and three-dimensional printed teeth models: a pilot study	Journal of Dental Education	8	2.67	0.89
Daud et al. (2023)	Enhancing learning experiences in pre-clinical restorative dentistry: the impact of virtual reality haptic simulators	BMC Medical Education	7	3.50	2.00
Aliaga et al. (2020)	Preclinical assessment methodology using a dental simulator during dental students’ first and third years	Journal of Oral Science	7	1.40	0.17
Patil et al. (2023)	Effectiveness of haptic feedback devices in preclinical training of dental students-a systematic review	BMC oral health	5	2.50	1.43
Ziane-Casenave et al. (2022)	Influence of practical and clinical experience on dexterity performance measured using haptic virtual reality simulator	European Journal Of Dental Education	5	1.67	0.56
Sheng et al. (2022)	Virtual versus jaw simulation in inlay preparation preclinical teaching: a randomised controlled trial	BMC Medical Education	4	1.33	0.44
Zhou et al. (2019)	Towards ar-assisted visualisation and guidance for imaging of dental decay	Healthcare Technology Letters	4	0.67	0.14
Bakker et al. (2017)	Effect of students’ determination of testing time on their test performance	European Journal of Dental Education	4	0.50	0.17
Bandiaky et al. (2024)	Impact of haptic simulators in preclinical dental education: a systematic review	Journal of Dental Education	3	3.00	2.25
Baechle et al. (2022)	Practice makes perfect? Association between students’ performance measures in an advanced dental simulation course	Journal of Dental Education	3	1.00	0.33
Yang et al. (2023)	How does dental students’ expertise influence their clinical performance and perceived task load in a virtual dental lab?	Journal of Computing in Higher Education	3	1.50	0.86
Huang et al. (2023)	Application of virtual reality and haptics system simodont in Chinese dental education: a scoping review	European Journal of Dental Education	2	1.00	0.57
Chu et al. (2023)	Mirror training device improves dental students’ performance on virtual simulation dental training system	BMC Medical Education	2	1.00	0.57
Urbankova et al. (2022)	A complex haptic exercise to predict pre-clinic operative dentistry performance: a prospective study	Journal of Dental Education	2	0.67	0.22
Wright (2017)	Drawing links within dental education	Technology Knowledge and Learning	2	0.25	0.09
Alhamad et al. (2024)	Taxonomic discordance of immersive realities in dentistry: a systematic scoping review	Journal of Dentistry	1	1.00	0.75
Dalanon (2023)	Multiplatform and cost-effective augmented reality model development in restorative dentistry	Journal of Dental Education	1	0.50	0.29
Azhari et al. (2024)	Integrating virtual-reality into restorative dentistry training: a study on the acceptability and effectiveness of tooth preparation simulations	Journal of Dental Education	0	0.00	0.00
Hamama et al. (2024)	Benefits of using virtual reality in cariology teaching	BMC Medical Education	0	0.00	0.00
Oguzhan et al. (2024)	Implementation of machine learning models as a quantitative evaluation tool for preclinical studies in dental education	Journal of Dental Education	0	0.00	0.00
Daud et al. (2024)	The impact of virtual reality haptic simulators in pre-clinical restorative dentistry: a qualitative enquiry into dental students’ perceptions	BMC Oral Health	0	0.00	0.00
Li et al. (2024)	The application of a virtual rubber dam isolation training system in dental preclinical education	Heliyon	0	0.00	0.00
Nassief et al. (2024)	Dental students’ perceptions of the use of two-dimensional and three-dimensional vision in dental education using a three-dimensional haptic simulator: a qualitative study	Journal of Dental Education	0	0.00	0.00
Yeslam et al. (2024)	Revolutionizing cad/cam-based restorative dental processes and materials with artificial intelligence: a concise narrative review	PeerJ	0	0.00	0.00
Al Ali et al. (2024)	The value of stereoscopic three-dimensional vision on dental students’ performance in a virtual reality simulator	Journal of Dental Education	0	0.00	0.00
Algarni et al. (2024)	The impact of virtual reality simulation on dental education: a systematic review of learning outcomes and student engagement	Journal of Dental Education	0	0.00	0.00
Joseph et al. (2023)	Distinguishing skill levels with haptic simulation in restorative dentistry: myth or reality?	European Journal of Dental Education	0	0.00	0.00

^a^Total citations based on Web of Science Core Collection database.

### Citation analysis

**Fig. 2:**
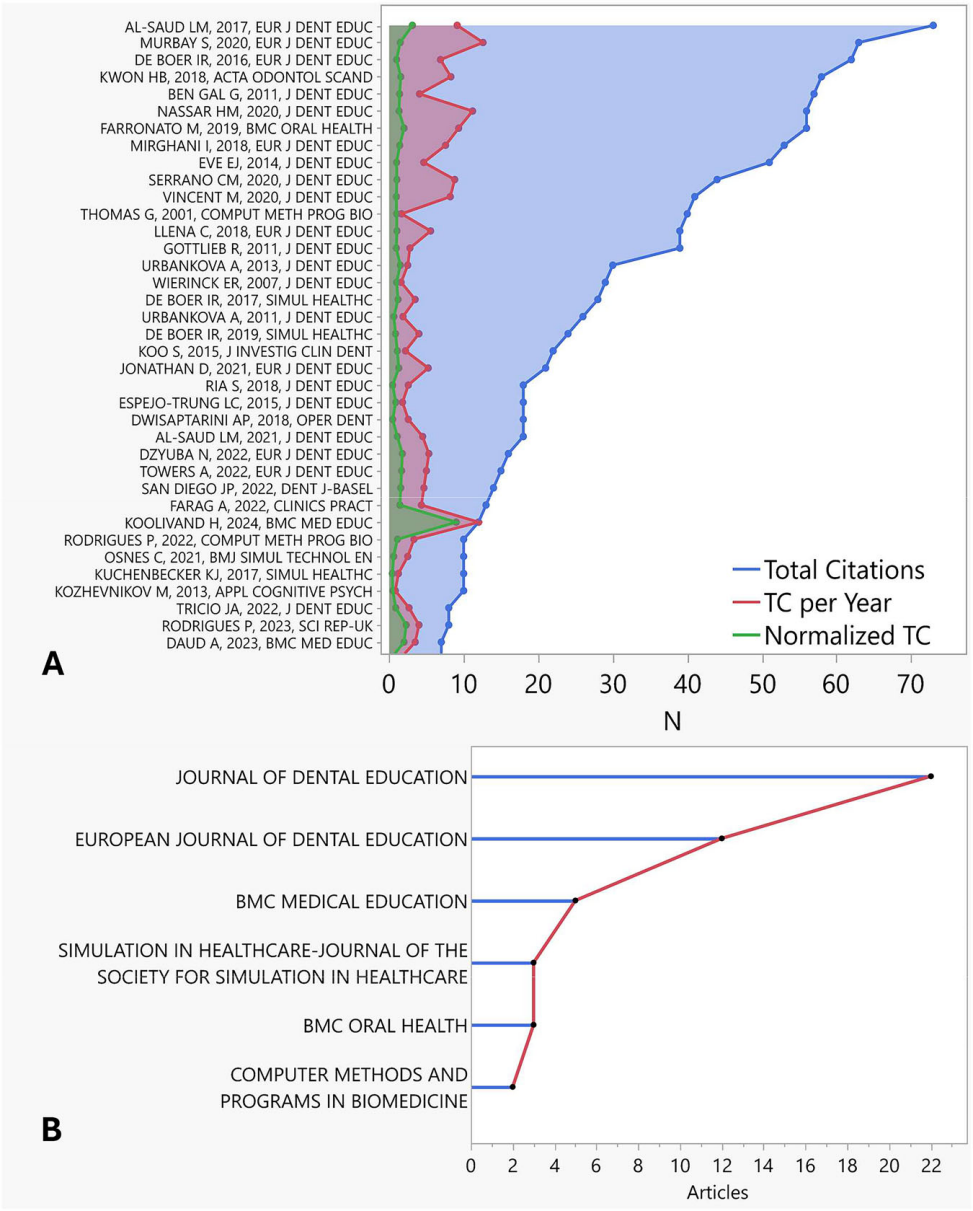
Citation metrics and journal contributions in virtual reality (VR) for restorative dentistry. **A** Line chart showing the total citations, total citations per year and normalized total citation for highest cited articles. **B** Top contributing journals related to VR in restorative dentistry.

Based on WOS-CC database, the top 37 most cited articles are presented in Fig. 2A. The most cited article in WOS-CC received a TC of 73 was titled “Feedback and motor skill acquisition using a haptic dental simulator” [22]. Based on WOS-CC the article was published in the *European Journal of Dental Education* in 2017 and attracted 9.13 TC per year and normalized TC of 3.12. The article is jointly affiliated with the University of Leeds (School of Dentistry and School of Psychology), Leeds, UK and College of Dentistry, King Saud University, Riyadh, Saudi Arabia.

The second most cited article, also published in the *European Journal of Dental Education*, attracted a total of 63 citations, was titled; “Evaluation of the introduction of a dental virtual simulator on the performance of undergraduate dental students in the pre-clinical operative dentistry course” [13]. In comparison with the above most cited study, Murbay et al. attracted a higher TC per year of 12.60 with a lower normalized TC of 1.49 and was solely affiliated to Faculty of Dentistry, The University of Hong Kong, Hong Kong.

The third most cited article, also published in the *European Journal of Dental Education* in year 2016, attracted a total of 62 citations was titled “student performance and appreciation using 3D vs. 2D vision in a virtual learning environment” [23]. The article attracted 6.89 TC per year, a normalized TC of 1.00 and solely affiliated to the Academic Centre for Dentistry Amsterdam (ACTA), University of Amsterdam and VU University Amsterdam, Amsterdam, the Netherlands.

### Journal analysis

Included articles were published in a total of 21 journals. Figure 2B illustrates the top six contributing journals and include; *Journal of Dental Education* (Impact Factor [IF] = 1.4), *European Journal of Dental Education* (IF = 1.7), *BMC Medical Education* (IF = 2.7), *Journal of The Society for Simulation in Healthcare* (IF = 1.7), *BMC Oral Health* (IF = 2.6), and *Computer Methods and Programs in Biomedicine* (IF = 4.9). The impact factor reported is based on the latest number from the journal citation report 2023.

### Year of publication

**Fig. 3:**
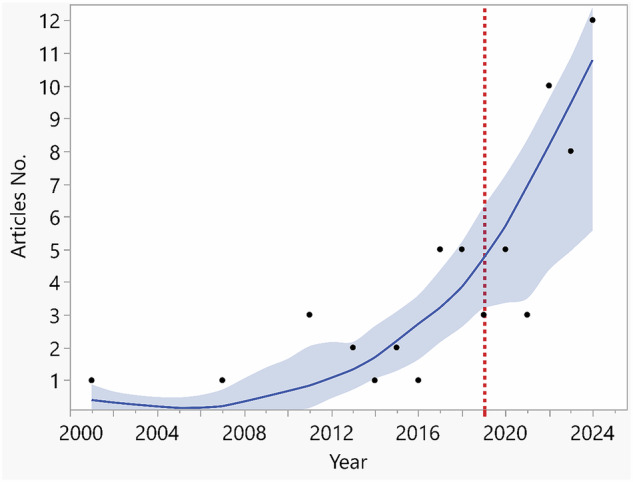
Line chart showing articles production over the past 20 years. The red dotted line in 2019 separate between pre-COVID and post-COVID era.

Since 2001, research articles investigating the use of VR in restorative dentistry just started to populate in the scientific literature. However, it was not until the year 2019 in which an apparent increase in publications focusing on VR in restorative dentistry took place. A marked surge was noted from the year 2021 to date and clearly illustrated in Fig. 3.

### Country cooperation analysis

**Fig. 4:**
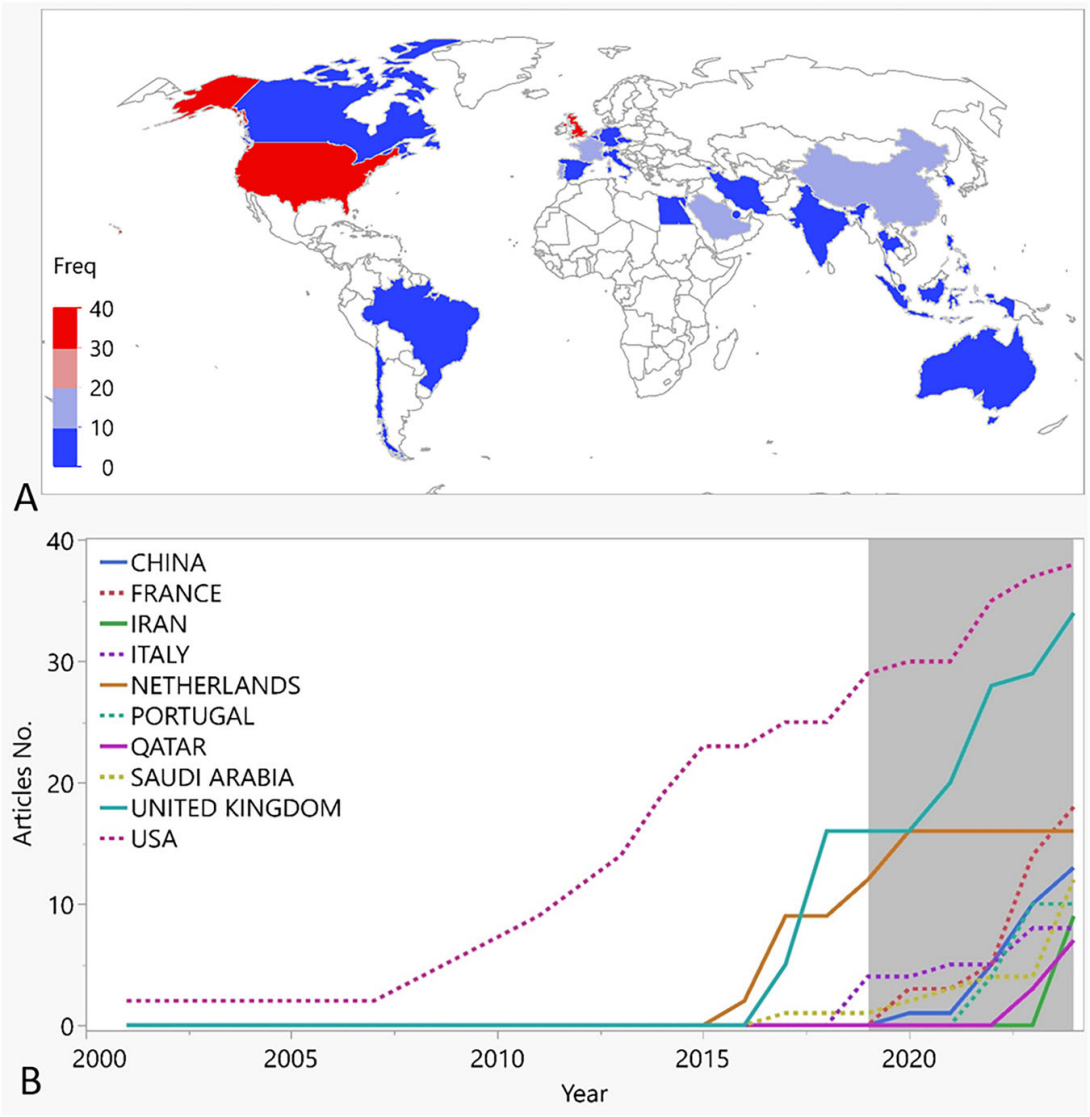
Geographic distribution and temporal trends in publication output. **A** Global map illustrating the country production count. Highest production countries are coded red as can be seen for the USA (*n* = 38) and UK (*n* = 34). **B** Line chart illustrating the top ten countries in publication output over time.

Authors from 27 countries participated in the analyzed articles as seen in Fig. 4. The top five most productive countries included; the USA (*n* = 38), UK (*n* = 34), France (*n* = 18), Netherlands (*n* = 16), and China (*n* = 13). The USA had an early start, with publications dating back to 2007. Interest in this topic grew significantly from 2015 onward, with the UK and the Netherlands emerging as leading contributors.

### Institution analysis

**Fig. 5:**
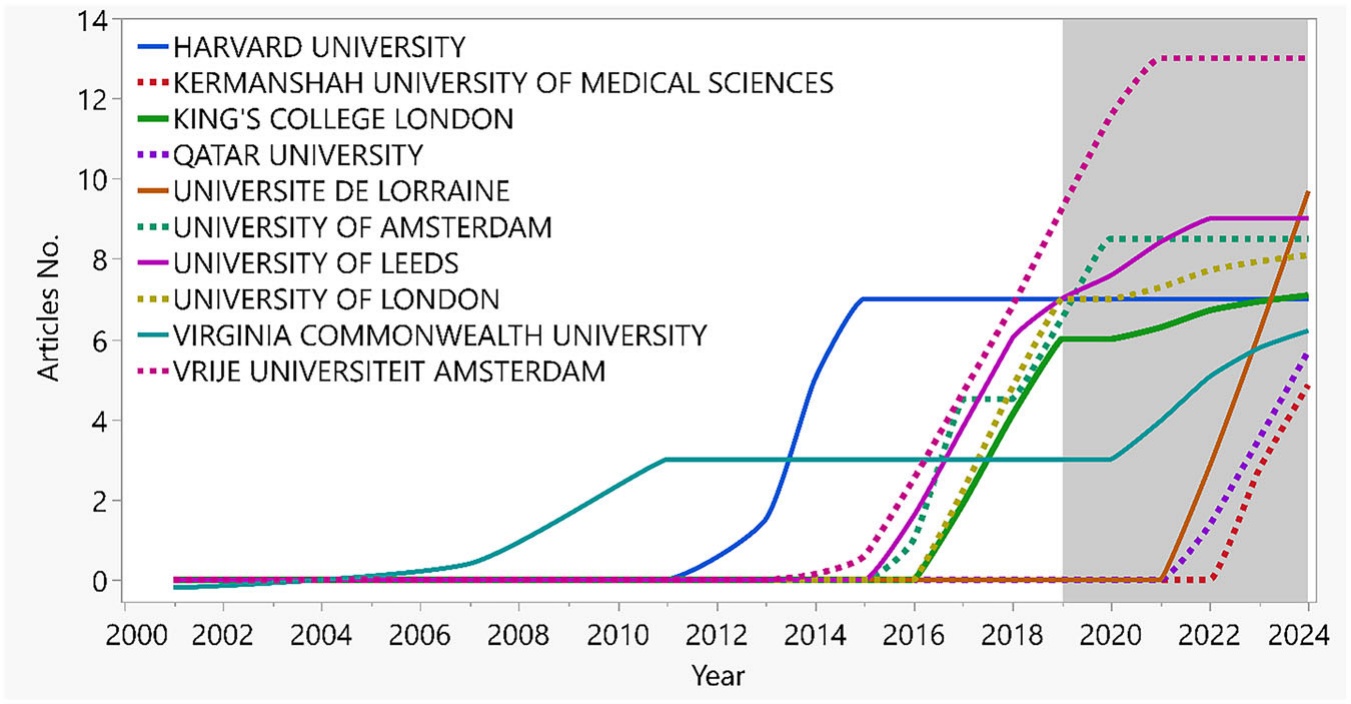
Line chart illustrates the highest ten affiliated institutes with production over time. The highest was for Vrije Universiteit Amsterdam (*n* = 13), followed by the Université de Lorraine (France), Academic Center for Dentistry Amsterdam (ACTA, Netherlands), and University of Leeds (UK) with 9 articles/each.

Included articles were produced by authors from 107 universities/institutes. The Vrije Universiteit Amsterdam (Netherlands) showed the highest article numbers (*n* = 13), followed by Université de Lorraine (France), Academic Center for Dentistry Amsterdam (ACTA, Netherlands), and University of Leeds (UK) with 9 articles/each. Harvard University (USA), University of Amsterdam (Netherlands), and University of London (UK) published 8 articles/each as seen in Fig. 5. It is worth mentioning that The Vrije Universiteit Amsterdam and University of Amsterdam merged under the ACTA [24], however the affiliations were distributed under the three names, as the authors were uncertain about the most up to date affiliation and was kept as per database extraction.

### Author analysis

**Fig. 6:**
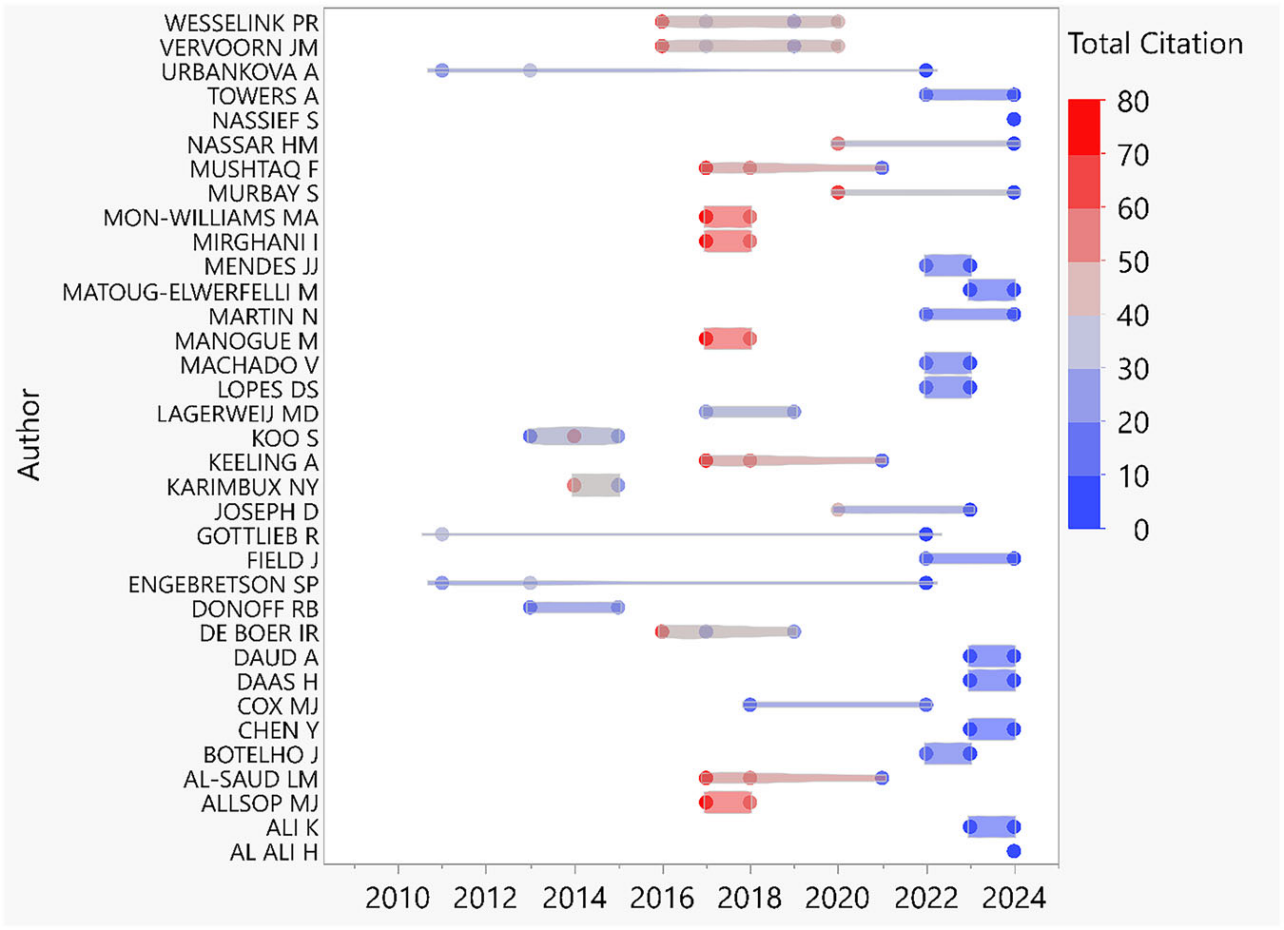
Author production over years. The total citation was color coded (red: higher and blue: lower). Only the early publications (2014–2018) showed a total higher citation count, while the publications from 2019 showed average citation count between (0–20).

**Fig. 7:**
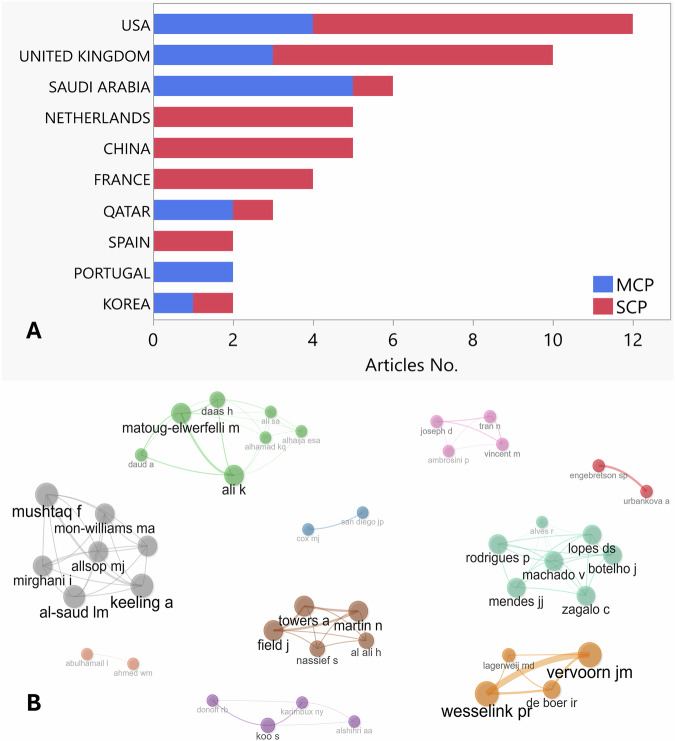
Author distribution and collaboration structure. **A** Geographic distribution of corresponding authors. MCP: Multiple country publication. SCP: Single country publication. **B** Authors collaboration network. Cluster analysis shows limited collaboration between different teams (as visualized with no connecting lines between different similar color clusters).

Results analysis revealed a total of 267 authors contributing to the entire 62 articles, with the author production over-year presented in Fig. 6. The average number of authors per article was 5.07, while only three articles were classified as single-authored articles [25–27]. The corresponding author/s countries are presented in Fig. 7A, with the USA ranking the country (*n* = 12). Authors collaboration network is illustrated in Fig. 7B and reveals limited collaboration between researches teams.

### Keywords co-occurrence map

**Fig. 8:**
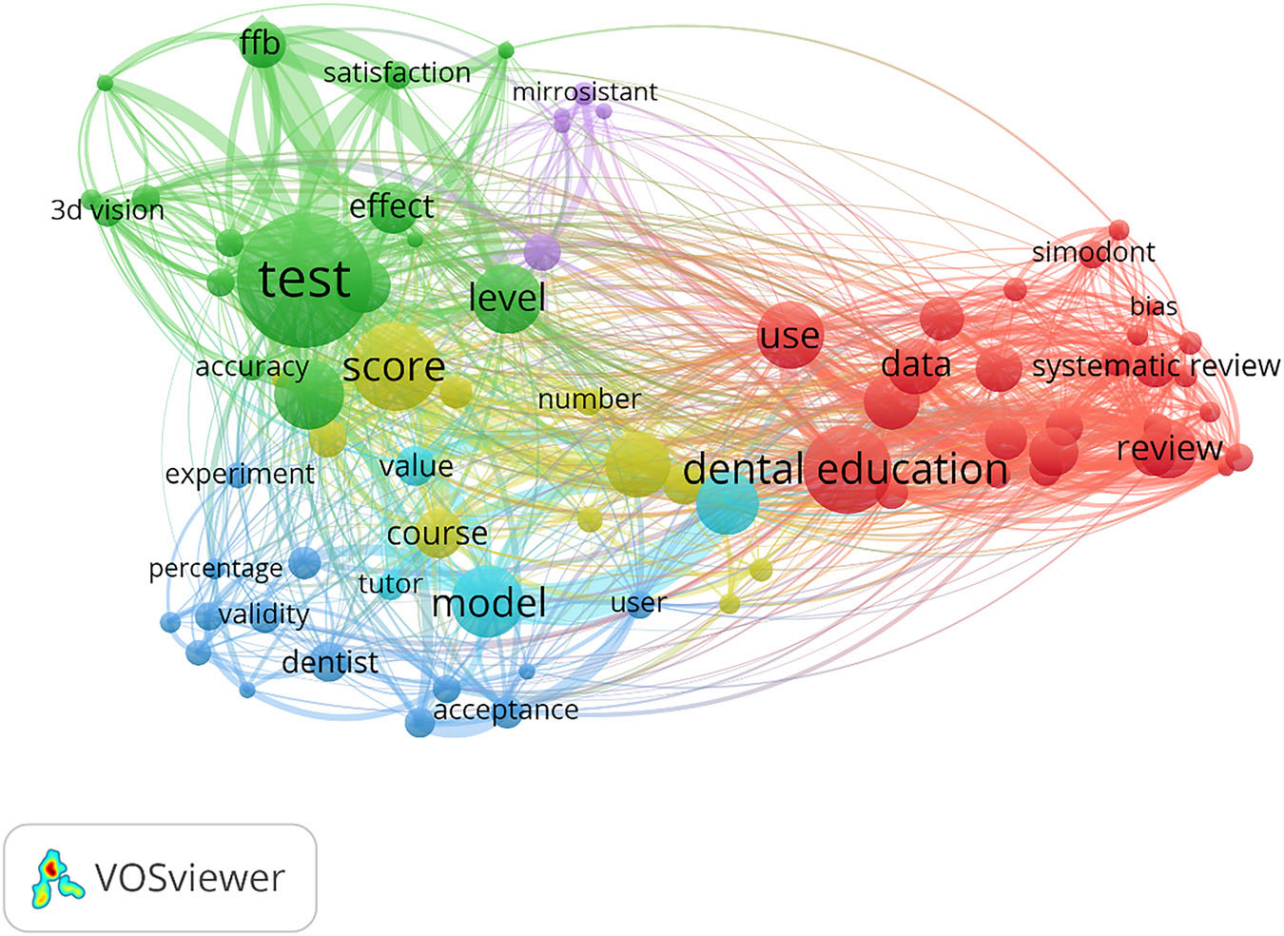
Network analysis of the most frequent keywords in the title and abstract of the included studies.

The keywords were automatically generated from the WoS searched database of the included studies using VOSviewer software for the generation of network visualization. The overview of the extracted words (from title and abstract) revealing interests of dental researchers and the frequency of their occurrence is illustrated in Fig. 8. In the network visualization, the size of the circles indicates the frequency of occurrence and the level of existing knowledge for each concept. The nodes with the same color indicate a word cluster, and the lines depict their relationships.

## Discussion

Clinical dental education relies heavily on development of fine motor skills and involves extensive pre-clinical laboratory training in simulated settings to ensure students achieve competency before transitioning to clinical practice [28]. Although successful, traditional mannequin teaching methods face several limitations, including the availability of natural teeth, the cumulative cost of plastic teeth and essential materials, and the inherent risks of novice students handling sharp instruments [22]. To overcome these challenges, alternative teaching strategies have been explored. Immersive technologies, a rapidly evolving field, offer novel educational and clinical applications. Within dentistry, immersive reality tools are gaining significant popularity, with a notable increase in the number of publications in the last five years [3].

This bibliometric analysis provides valuable insights into the evolving research landscape on VR in restorative dentistry. By examining publication trends, authorship patterns, journal impact, and collaboration networks, this study highlights the increasing integration of VR into dental education. The findings reflect a growing interest in VR-driven training, aimed at enhancing hand-eye coordination and procedural skills among dental students. Similar trends have been reported in other dental disciplines, such as endodontics [29, 30], and dental traumatology [20], where bibliometric studies have observed rising research outputs in recent years.

In this study, the WoS-CC database was utilized as a standard reference for citation analysis, as it is widely recognized for tracking scientific article citations across an extensive timeframe [29, 31, 32]. A total of 62 articles met the inclusion criteria, revealing a significant increase in VR-related publications in restorative dentistry, particularly after 2019. This surge may be linked to the COVID-19 pandemic, which accelerated the adoption of digital and remote learning tools. This trend aligns with previous bibliometric studies in dentistry, which have noted a shift toward technology-driven research in response to educational and clinical challenges [29].

Among the journals publishing VR-related studies in restorative dentistry, the *Journal of Dental Education* had the highest number of articles (*n* = 22). Established in 1936 and published by John Wiley & Sons, it remains a leading journal in oral health education, with a 2023 Journal IF of 1.4. However, it was indexed in WoS only from year 2009, leading to the exclusion of VR-related studies published earlier. Notable excluded work includes Buchanan (2004), affiliated with the University of Pennsylvania, which explored VR-based teaching in restorative procedures [33]. Similarly, the *European Journal of Dental Education* was indexed in WoS only from 2009, leading to the omission of some studies. Notable among these were two studies by Quinn et al. in 2003 affiliated to Dublin Dental School, which compared conventional and VR-based training for junior dental students [34, 35]. Additionally, two studies by Wierinck et al. conducted in 2005 and 2006 from Leuven, Belgium, explored the impact of augmented feedback on manual dexterity training. Their findings indicated that frequent feedback in VR-based Class I cavity preparation significantly enhanced novice students’ long-term learning outcomes [36, 37].

In this study, the citation analysis revealed that the most influential articles, based on TC per year, were those emphasizing the practical application of VR technologies in dental training. The article by Murbay et al., which evaluated the impact of a dental virtual simulator on undergraduate performance, ranked highest with 12.60 citations per year, despite having a lower normalized citation rate [13]. This was followed closely by Koolivand et al., a recent systematic review comparing virtual reality and conventional teaching, also receiving 12.00 citations per year, despite being newly published [38]. In third place was Nassar et al., which offered a critical review of simulation-based education, earning 11.20 citations per year [39]. These studies underscore the growing demand for evidence-based evaluations of VR in dental education, with strong citation performance suggesting both academic influence and relevance to pedagogical practice (Table 1).

The findings highlight a clear trend: articles with the highest citations per year tend to explore not only the technological capabilities of VR, such as haptic feedback and immersive simulations, but also their direct implications for student performance, confidence, and educational outcomes. This aligns with broader trends in health professions education, where the integration of technology is increasingly assessed through learner-centric outcomes. These metrics support the assertion that immersive VR environments enhance psychomotor skill development and reduce anxiety during skill acquisition, contributing to increased student engagement and competence.

This bibliometric analysis indicates that the UK and USA are the leading contributors to VR research in restorative dentistry. The analysis also shows significant contributions from institutions in China, France, and the Netherlands, reflecting the international interest in this emerging field. The USA demonstrated an early interest in this field, with publications dating back to 2007, primarily affiliated with Harvard University. In contrast, the UK, with key contributions from the University of Leeds, and universities across the Netherlands, showed a notable increase in research activity around 2015. France, with significant contributions from the University of Lorraine, ranked third in overall output. These findings underscore the global interest in VR applications in restorative dentistry, with research hubs emerging across multiple regions over time. However, the collaboration network analysis illustrated limited interdisciplinary and international collaborations in this field of research (as seen in Fig. 7 with no connecting lines between different clusters). Further collaboration between institutes and countries is recommended to further enhance research productivity and impact in this domain. For instance, partnerships between dental schools and technology-focused institutions have facilitated the development and evaluation of innovative VR tools for dental education [14, 40].

Overall, simulation training using VR technologies has been extensively studied for its potential to replicate real-life clinical scenarios in a controlled environment. VR-based simulators with haptic feedback can improve students’ manual dexterity and procedural accuracy [10]. The use of virtual patient scenarios in restorative dentistry has also been reported to allow students to practice diagnostic and treatment planning skills in a risk-free environment [5]. These scenarios have been shown to improve students’ critical thinking and decision-making abilities, which are essential for clinical practice [13]. Indeed, the integration of VR technology in dental curricula has been recommended by several studies to bridge the gap between theoretical knowledge and clinical practice [22].

### Research gaps and future directions

Despite the growing body of research on VR in restorative dentistry, several gaps remain. For instance, there is a lack of longitudinal studies evaluating the long-term impact of VR training on students’ clinical performance and patient outcomes. Additionally, more research is needed to assess the cost-effectiveness and scalability of VR technologies in dental education. Future research should also explore the integration of augmented reality (AR) and mixed reality (MR) technologies, which offer additional layers of interactivity and immersion. These technologies have the potential to further enhance the learning experience by providing real-time feedback and guidance during simulated procedures [3].

### Implications for dental education and practice

The growing interest in VR technologies reflects a broader shift towards digital and remote learning solutions in response to evolving educational needs and challenges. Dental schools may consider incorporating VR-based training tools into their curricula to enhance students’ clinical skills and preparedness for real-world practice. The adoption of VR technologies in clinical practice can improve patient safety and outcomes by allowing students to refine their skills in a simulated environment before performing procedures on actual patients. This aligns with the broader goals of competency-based education and patient-centered care in dentistry.

### Limitations of the study

While this bibliometric analysis provides valuable insights into the research landscape of VR in restorative dentistry, it is not without limitations. The analysis is based solely on data from the WoS-CC, which may not capture all relevant publications in this field. The WoS-CC was selected due to its rigorous indexing standards and strong citation tracking. The using a single, high-quality source ensured consistency and minimised the risk of duplication or metadata inconsistencies [41]. Future bibliometric studies should consider using multiple databases and incorporating altimetric indicators to provide a more comprehensive view of research impact. This would help to capture the broader influence of VR research on dental education. The use of VR in restorative dentistry has shown significant interest which is reflected in the publication of high number of scholarly papers.

## Conclusion

This bibliometric analysis highlights the growing interest in VR technologies within restorative dentistry and their potential to transform dental education and clinical practice. While significant literature is available, more research is needed to address existing gaps and explore the full potential of VR and other immersive technologies in dentistry. Additionally, researchers should focus on interdisciplinary and international collaborations in driving research productivity and enhancing the overall impact of advancements in this field.

## Data Availability

The data that supports the findings of this study are available on request from the corresponding author.
